# Increasing evidence of tick-borne encephalitis (TBE) virus transmission, the Netherlands, June 2016

**DOI:** 10.2807/1560-7917.ES.2017.22.11.30482

**Published:** 2017-03-16

**Authors:** Adriaan CG Weststrate, Daan Knapen, Gozewijn D Laverman, Bart Schot, Jan JW Prick, Silke A Spit, Johan Reimerink, Barry Rockx, Felix Geeraedts

**Affiliations:** 1 Department of Internal Medicine, Ziekenhuis Groep Twente, Almelo/Hengelo, the Netherlands; 2 Department of Neurology, Ziekenhuis Groep Twente, Almelo/Hengelo, the Netherlands; 3 Public Health Service, Twente, the Netherlands; 4 Center for Infectious Disease Control, National Institute for Public Health and the Environment (RIVM), Bilthoven, the Netherlands; 5 Laboratory for Medical Microbiology and Public Health, Hengelo, the Netherlands

**Keywords:** The Netherlands, vector-borne infections, zoonotic infections, viral infections, emerging or re-emerging diseases, tick-borne diseases, tick-borne encephalitis - TBE, Flaviviridae

## Abstract

We present a case of endemic tick-borne encephalitis (TBE) occurring in June 2016 in the eastern part of the Netherlands in an area where a strain of TBE virus, genetically different from the common TBE virus strains in Europe, was reported in ticks in 2016. With the start of the tick season in spring, this second autochthonous Dutch TBE case should remind physicians to consider the possibility of endemic TBE in patients with respective symptoms.

We report the second autochthonous human case of tick-borne encephalitis (TBE) ever diagnosed in the Netherlands in an area where a strain of TBE virus, genetically different from the common TBE virus strains in Europe, was identified in ticks in 2016.

## Case description

On 14 July 2016 (day 1), a 44 year-old male patient was admitted to the hospital with tinnitus, malaise, vomiting, muscle aches, and headache, that had started on 8 July. Shortly before symptom onset, starting on 25 June, he had also experienced a short episode of malaise and fever, from which he recovered spontaneously. A week before the first symptoms, he had been bitten by a tick, without having developed erythema migrans. The patient lives in the Sallandse heuvelrug area in the eastern part of the Netherlands, where tick-borne encephalitis virus (TBEV) had been detected in ticks in 2016 [1]. He maintains a garden in which he frequently encountered ticks. He also reported frequent outdoor activities such as running and walking in the area where he lived. Six weeks before the first symptoms, he had travelled to Bad Salzungen in Thuringia, Germany, for 2 days, but did not reside in a wooden area, and was not bitten by ticks. He was vaccinated against yellow fever in 1996 but not vaccinated against TBEV. The patient did not report any consumption of unpasteurized milk products in the month before illness onset.

Physical examination revealed no abnormalities. Laboratory tests showed signs of inflammation: C-reactive protein (CRP): 45 mg/L (norm: <10 mg/L); leukocytes: 10.4 x 10^9^/L (norm: 4–10 mmol/L). A slightly lower haemoglobin (Hb) concentration (7.8 mmol/L; norm: 8.5–11.0 mmol/L) possibly indicative of an infection ongoing for some time. The result of the thorax X-ray examination was normal.

The dehydrated patient was hospitalised with differential diagnosis being gastroenteritis, borreliosis, or a bacterial infection. Ceftriaxone therapy intravenously (2 g per day) was initiated. On day 2, his condition did not improve, and an overt mental slowness became evident as well as a tremor in both hands, with concurrent loss of strength.

Lumbar puncture was performed and cerebrospinal fluid (CSF) analysis revealed leukocytosis (99 x 10^6^/L (norm: <5 x 10^6^/L); 85% mononuclear cells; 15% polynuclear cells (norm: <6%); glucose and protein concentrations of 3.3 mmol/L (norm: 2.2–3.9 mmol/L) and 1,101 mg/L (norm: 250-800 mg/L) respectively. The working diagnosis now was a meningoencephalitis, possibly caused by a virus, or neuroborreliosis. Acyclovir, intravenously 3 mg/kg, 3 times per day, was added to the treatment regimen.

ELISA tests on both CSF and serum did not elicit Borrelia-specific antibodies. Nucleic acid detection of various potential pathogens in the CSF turned out negative (Table 1). Due to a report by the Dutch National Institute for Public Health and the Environment (RIVM), on 30 June 2016, of the first time detection of TBEV in ticks in the Sallandse heuvelrug region [1], serum was investigated for TBEV-specific antibodies. Pending the results, doxycycline, 100 mg twice per day, was added to the regimen, because rickettsiosis could not be excluded at the time. A brain magnetic resonance imaging (MRI) was performed, showing an enlargement and staining of the dura mater, which may suggest viral meningitis.

**Table 1:** Nucleic acid detection of pathogens in cerebrospinal fluid, tick-borne encephalitis (TBE) case, the Netherlands, June 2016

Pathogen	PCR result
*Borrellia burgdorferi*	Negative
*Mycoplasma pneumoniae*	Negative
*Haemophilus influenzae*	Negative
*Neisseria meningitidis*	Negative
*Streptococcus agalactiae*	Negative
*Streptococcus pneumoniae*	Negative
Herpes simplex virus	Negative
Varicella zoster virus	Negative
Adenovirus	Negative
Enterovirus	Negative
Human parechovirus	Negative

On day 7, the results of the TBEV serology confirmed the following: anti-TBEV IgM and IgG were detected in serum with the ELISA method, suggesting a recent TBEV infection (Table 2). Also, the CSF turned out positive for anti-TBEV IgG and borderline positive for anti-TBEV IgM. The antibiotics and acyclovir were stopped. By day 9 the patient gradually improved. However, the tinnitus persisted.

**Table 2:** Tick-borne encephalitis (TBE) virus diagnostic test results, TBE case, the Netherlands, June 2016

	Material	CSF	Serum	Serum	Urine
	Date (2016)	15 July	15 July	8 August	8 August
RIVM	Serology (ELISA)	IgM borderline positive OD _450_ 0.421	IgM positive 355 VIEU/mL	IgM positive 341 VIEU/mL	NA
		IgG positive OD _450_ 3.064	IgG positive > 650 VIEU/mL	IgG positive > 650 VIEU/mL	
	PCR	Negative	ND	ND	Negative
Medical University, Vienna	Serology (ELISA)	ND	IgM positive	IgM positive	NA
			IgG positive > 1,000 VIEU/mL	IgG positive 50,600 VIEU/mL	
	Virus neutralisation assay	ND	Positive titre: 1:10	Positive titre: 1:480	NA

CSF: cerebrospinal fluid; NA: not applicable; ND: not done; OD^450^ Optical density at 450 nm (IgM norm: <0.360 (negative); IgG norm: <0.270 (negative); RIVM: Rijksinstituut voor Volksgezondheid en Milieu (National Institute for Public Health and the Environment); VIEU: Vienna Units.

The ELISA results were confirmed with a virus neutralisation assay, by the Department of Virology of the Medical University Vienna, Austria. A second serum sample taken on 8 August showed an increase in virus neutralising antibody titres (from 1:10 to 1:480), which is proof of an acute infection.

## Background

TBE is a zoonotic infectious disease, caused by TBEV. Natural hosts are small rodents while larger mammals like deer and humans may become accidentally infected. Infection of humans mainly occurs by transmission of the virus through the bite of an infected tick (*Ixodes* species). The chance of transmission depends on the activity of ticks, and increases during spring. Alternatively, infection may occur by ingestion of milk and milk products from viraemic ruminants [2]. TBE caused by the European subtype TBEV (TBEV-Eu) characteristically has a biphasic course, starting with influenza-like symptoms such as fever, malaise, headache and myalgia (average 2–7 days). After a successive symptom-free interval (average 2–10 days), approximately one third of the cases develop neurological symptoms, varying from mild meningitis to severe encephalitis, with or without myelitis and paralysis of extremities and breathing muscles [3,4].

In Europe, areas of risk for TBE are predominantly situated in central and eastern Europe, and the Baltic and northern countries [5]. In the Netherlands, TBE was only considered as an imported disease until the first case of autochthonous TBE was reported, occurring in June 2016, shortly before our case [6]. In the first case, the virus was recovered and showed strong homology with the common TBEV-Eu strains that cause disease in Europe. The infection likely occurred in a forested area between Driebergen en Maarn, in the province of Utrecht. There was no link to the Sallandse heuvelrug region, in the province of Overijssel, which was at that time the only region in the Netherlands known to harbour TBEV infected ticks. There are currently no proven infections described with the TBE-Eu strain of the Sallandse heuvelrug, which is genetically distinct from the common TBE-Eu strains (data not shown and [6]).

## Discussion

We present a confirmed case of TBE, according to the European Union case definition (symptoms of meningoencephalitis combined with a rise in TBEV-specific antibody titres) [7]. The typical biphasic clinical course in our case fits the diagnosis TBE. Vomiting, a prominent symptom in our case, is usually not described in association with TBE, nor is tinnitus [3,4].

The microbiological diagnosis of TBE is based on serological tests. Because of the strong likelihood of cross reactivity with antibodies against TBEV-related viruses, other flaviviruses, as for instance yellow fever virus, positive ELISA results need to be confirmed with a virus neutralisation assay [8]. In this way, confirmed ELISA results combined with a rise in TBEV-specific antibody titres, make a different explanation for the positive test results, as for instance a past yellow fever vaccination, unlikely.

Chances are high that the TBEV infection was acquired in the Sallandse heuvelrug region. The patient was bitten by a tick in that region during the incubation phase, which ranges from 4 to 28 days (average 7–10 days) [4], and he had neither been in the Utrechtse heuvelrug region, nor in a TBEV-endemic area abroad. His visit to Bad Salzungen was two weeks before the maximum incubation time of 28 days, it lasted only shortly, and was without at-risk activities. Moreover, Bad Salzungen is not regarded one of the risk areas in Germany [9].

For a definite proof that the infection was caused by the TBEV-Eu strain from the Sallandse heuvelrug, nucleic acid sequence analysis of the virus, recovered from the patient or tick, would be required. This is of clinical importance, because the TBEV-Eu strain from the Sallandse heuvelrug genetically diverges from the commonly found TBE-Eu strains, and it is not known whether this strain infects people and causes symptoms. There is 93% homology between the Sallandse heuvelrug TBEV-Eu strain and the prototype TBEV-Eu Neudörfl strain (data not shown), which is found in large parts of Europe. For comparison, the TBEV-Eu strain from the first Dutch TBE case, reported in August 2016, showed 99% homology with this common TBEV-Eu strain [6].

Recovering of nucleic acid sequences was, however, not possible. The aforementioned tick had not been saved, and the chance of detecting the virus in patient materials at the time of neurological symptoms is extremely small [8] (also Table 2). Based on the place of residence and travel history of the patient, it seems however likely that the acquired infection was caused by the Sallandse heuvelrug TBEV-Eu strain because this strain represents the single strain that has been found in this region so far (data not shown).

Although a large national survey is lacking, based on the current knowledge, the percentage of TBEV infected ticks seems to be low (< 0.1% in the Sallandse heuvelrug region). In comparison, the average percentage of ticks in the Netherlands infected with *Borrelia burgdorferi* was estimated between 15 and 20% [2]. Besides *Borrelia burgdorferi* and TBEV, ticks in the Netherlands have been shown to harbour other potential human pathogens, like *Anaplasma*, *Rickettsia*, *Candidatus Neoehrlichia*, *Babesia* spp., and *Borrelia miyamotoi*, but their clinical relevance is still unclear [10].

Worldwide, TBEV infections have been on the rise in recent years, and endemic areas have been expanding [11]. When and from where TBEV was introduced into the Sallandse heuvelrug area is unclear. Deer from this region were shown to carry TBEV-specific antibodies, in a survey dating from 2010 (data not shown). It is therefore possible that TBEV has been present in the Netherlands for already some years, and TBEV infections may have been overlooked. The majority of TBEV infections do not lead to TBE symptoms. This suggests that for the two identified autochthonous TBE cases in the Netherlands, a larger number of (undiagnosed) TBEV infections might be expected.

Currently, no specific therapy that targets TBEV exists. Effective vaccines against TBE are available, and are on the market since the 1980s. No advice on TBE vaccination exists for the Netherlands [2]. The risk of TBEV infection in the Netherlands is currently being assessed by the RIVM.

## Conclusion

This is the first described case of TBE associated with the Sallandse heuvelrug region, where TBEV is endemic in ticks. Yet, definite proof that the infection was caused by the specific TBEV-Eu strain from that region is lacking. Being the second autochthonous TBE case in the Netherlands, however, it provides more evidence for endemic TBEV transmission in this country. With the new tick season approaching, clinicians in the Netherlands should consider TBE in the differential diagnosis of patients with meningoencephalitis symptoms especially after a tick bite, even if there has been no recent travel to a known TBE-endemic country.
